# Vertebral intraosseous plasma rich in growth factor (PRGF-Endoret) infiltrations as a novel strategy for the treatment of degenerative lesions of endplate in lumbar pathology: description of technique and case presentation

**DOI:** 10.1186/s13018-020-01605-w

**Published:** 2020-02-24

**Authors:** Fernando Kirchner, Ariadna Pinar, Isidro Milani, Roberto Prado, Sabino Padilla, Eduardo Anitua

**Affiliations:** 1 Barcelona Traumatology Institute, Mataró, Spain; 2 Eduardo Anitua Foundation for Biomedical Research, Jacinto Quincoces, 39, 01007 Vitoria, Spain; 3 BTI-Biotechnology Institute ImasD, Vitoria, Spain

## Abstract

**Background:**

Motivation and necessity to adopt minimally invasive therapies in the field of spinal regenerative medicine is increasing. Autologous platelet-rich plasma (PRP) therapy has recently been used as an effective technological and biological approach to tissue repair and has shown to improve multiple conditions including back pain and degenerative disc pathology. In addition, it is well established that the anatomic elements of the spinal system affected by degenerative pathology include the intervertebral disc (IVD) and vertebral subchondral bone (VSB), which play a crucial role in maintaining a healthy spinal column. Both elements are the target of a novel biological approach to the treatment of low back pain.

**Methods:**

A novel minimally invasive regenerative therapeutic approach is presented herein with a protocol based on combining vertebral intraosseous (VIO) and intradiscal (ID) infiltrations of plasma rich in growth factors (PRGF-Endoret), a type of leukocyte-free PRP, for the treatment of disc degeneration pathology.

**Results:**

We describe a novel technique applied in a patient treated for IVD degeneration and VSB damage, showing significant improvement on magnetic resonance imaging, including partial regression of protruded disc and significant resorption of intravertebral herniations (Schmörl’s nodes), after PRGF therapy.

**Conclusions:**

To the best of our knowledge, we present the first reported case description of the utilization of VIO and ID PRP infiltrations to treat protruded discs and intravertebral herniations with a successful clinical outcome.

## Background

Prevention and treatment of low back pain (LBP) is a challenge in public health programs [1]. According to the latest data, LBP presents the world’s highest burden of disease related to years lived with disability [2] and is globally the fourth cause of disability-adjusted life years [3].

Classical surgical intervention and palliative treatments have been widely used for years to treat lumbar spine pathology, based on patients’ clinical symptoms. Nevertheless, nowadays, there is a consensus in the medical community regarding the importance of implementing minimally invasive techniques for lumbar disc degenerative disease (DDD), especially biological therapies [4].

Intervertebral discs (IVDs) and vertebral subchondral bone (VSB) are important anatomical elements of the spinal column affected by pain and degenerative pathology. Healthy IVDs provide stable support to contiguous spinal vertebrae and permit painless movement of the vertebral bodies, thereby contributing to spine flexibility. Indeed, the whole can be considered as an intervertebral joint functional unit, composed of an IVD, the upper and lower vertebrae, and the facet joints [5]. In adults, an endplate bilayer of the cartilage (CEP) and bone (VSB) is located at the ends of each IVD, separating the vertebral bone from the IVD itself and preventing the central, gel-like, hydrated nucleus pulposus from bulging outward into the neighboring spinal canal and nerves. In contrast to IVDs, the central endplate (EP) of the VSB is well innervated, as is the adjacent vertebral marrow. It is known that the VSB plays an important role in spinal function, maintaining IVD integrity and disc nutritional supply. Changes in IVD and VSB biomechanical and biochemical properties are associated with the development of back pain and DDD [6]. Some structural VSB alterations have been linked to disc degeneration, including acute changes detected by axial and sagittal T2-weighted magnetic resonance imaging (MRI), such as Schmörl’s nodes (SN) [7]. SNs are the herniation of nucleus pulposus of the IVD through the EP into an adjacent vertebral body [8]. Most SNs are asymptomatic, without pain, although some have been shown to become painful and to correlate with inflammation or edema of the vertebral body in patients with back pain [7]. SNs have also been correlated with Modic changes, which correspond to MRI changes in the vertebral-body bone marrow associated with DDD. Symptomatic SNs are mainly treated with conservative therapy (analgesics, non-steroidal anti-inflammatory drugs, corticosteroids, and tumor necrosis factor alpha (TNF-α) inhibitors) and surgery (vertebroplasty and lumbar fusion) [7].

Infiltrations of autologous plasma rich in growth factors (PRGF) have been widely used as an effective technological and biological approach to induce tissue repair and improve numerous clinical conditions [9]. Over the past few years, PRP has been included in techniques applied to specific spinal structures for the treatment of lumbar spinal pain associated with degenerative disc pathology and osteoarthritis [10–17].

We propose a novel minimally invasive regenerative approach to treat lumbar disc degenerative pathology based on two key principles: first, the physiological and structural roles of IVD and VSB in spinal function and degenerative pathology, and second, the great similarity with the intraosseous PRGF infiltrations that has been described in patients with knee [18] and hip [19] osteoarthritis for the treatment of chronic pain [20]. The combined infiltration of intradiscal [13] and vertebral intraosseous PRP was used to stimulate regeneration of the damaged spinal structures [5]. In such a way, also treating EP lesions, the IVD will regenerate earlier and faster, since the supply of nutrients to the IVD originates from the EP [6]. IVDs and VSB changes over time were assessed by MRI to monitor structural and functional changes of the spine.

## Methods

### Patient presentation

The treatment was undertaken after receiving the patient’s informed consent and in conformance with the international standards from the latest revised World Medical Association Declaration of Helsinki (Brazil, 2013) [21].

A 40-year-old man with a 10-year history of lumbar pain and failure of pain management was treated in a private orthopedics clinic. Medical examination elicited radiating pain in the left lower extremity.

Pain and function baseline information was obtained from the patient before the treatment. The values obtained were the following: NRS (Numeric Rating Scale) 9, COMI (The Core Outcome Measures Index) Score 6.85, and Oswestry Disability Index (ODI) 36% (moderate disability).

**Fig. 1:**
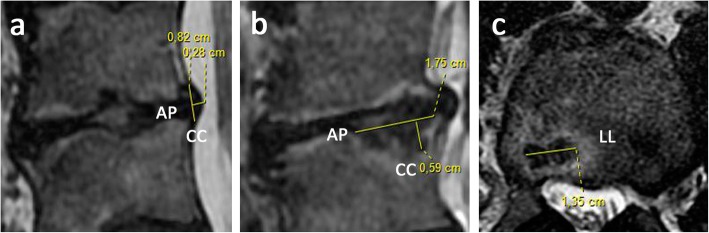
Axial and sagittal T2-weighted images (T2WI) pre-treatment at the lumbar spinal level L3/L4 showing MRI measurements (in mm). **a** Sagittal measurements of lumbar disc protrusion diameter at cranio-caudal (CC) and antero-posterior (AP) sections; **b** sagittal dimension of Schmörl’s node diameter (SND) at cranio-caudal (CC) and antero-posterior (AP) sections; and **c** axial dimension of SND at latero-lateral (LL) section

Lumbar MRI (Fig. 1) revealed several spinal alterations, including decreased T2-weighted (T2W) disc signal, indicative of degeneration; osteochondrosis; global disc dehydration; Modic changes (MC) [22]; intravertebral herniations or Schmörl’s nodes [22] and other types of injuries [23]; foraminal stenosis at the L5 level; and moderate and asymmetric disc impingements, primarily postero-paramedial (PP) herniated discs.

At the lumbar spinal level L3/L4 (Table 1), endplate degeneration (total endplate damage score (TEPS) 5) [24] and type 1 Modic changes were found, with Pfirrmann grade V disc degeneration (DD) [22]. Significant protrusion at the L3/L4 disc space causing compression of the L3 nerve root, and L3-lower and L4-upper SNs, particularly marked at the L4 level, were also found.

**Table 1:** A pre- and post-treatment magnetic resonance imaging analyses performed at the L3/L4 disc space and corresponding vertebral level

Spine L3–L4 level	MRI pre-PRGF	MRI post-PRGF
Protrusion diameter (PD)	mm	mm
Cranio-caudal (CC)	8.2	7.3
Antero-posterior (AP)	2.8	2.8
Discal degeneration degree	Grade	Grade
Pfirrmann classification	V	V
Endplate degeneration degree	Grade	Grade
Modic changes	1	1
Total endplate score (TEPS)	5	5
Schmörl’s node diameter (SND)		
L3-lower SND	mm	mm
Cranio-caudal (CC)	2.6	1.5
Latero-lateral (LL)	3.0	Unmeasurable
Antero-posterior (AP)	7.6	3.0
L4-upper SND	mm	mm
Cranio-caudal (CC)	5.9	4.5
Latero-lateral (LL)	13.5	11.5
Antero-posterior (AP)	17.5	17.0

According to axial and sagittal T2-weighted images (T2WI) of the lumbar spine, the networked medical imaging remote Workstation AW 4.3 (GE Healthcare International) was used to import, interpret, and process DICOM images from MRI scans to categorize and obtain measurements of the following MRI parameters: protrusion diameter (PD), disc degeneration degree (Pfirrmann classification), endplate degeneration degree (Modic changes and total endplate score), and Schmörl’s node diameter (SND)

Due to the complexity of the patient’s symptoms and the degree of disc degeneration and vertebral damage, it was proposed to carry out a novel biological approach: a combination of vertebral intraosseous infiltrations via the spinal percutaneous transpedicular biopsy method [25] and a previously described spinal PRGF therapy regarding the disc (epidural, articular, and intradiscal) [13].

### Platelet-rich plasma preparation

The platelet-rich plasma was prepared according to the PRGF-Endoret method [26]. Briefly, after informed consent was obtained, 72 ml of peripheral venous blood was withdrawn from the patient and collected in eight 9-ml tubes containing sodium citrate (3.8% wt/vol) as anticoagulant (Endoret Traumatology kit, BTI Biotechnology Institute, S.L., Vitoria, Álava, Spain). Then, the blood tubes were centrifuged at 580*g* for 8 min at room temperature in the PRGF-Endoret System IV centrifuge (BTI Biotechnology Institute, S.L., Vitoria, Álava, Spain). The upper plasma volume (F1) containing a similar number of platelets as peripheral blood was drawn off. The 2 ml plasma fraction (F2) located just above the buffy coat and red cells were collected in fractionation tubes without aspirating the buffy coat. This plasma fraction (F2) presents a moderate enrichment in platelets (approximately twofold the platelet count of peripheral blood) with scarce leukocytes. Prior to the infiltration, the PRGF (F2 fraction) was first activated by adding 20 μl PRGF activator (10% wt/vol calcium chloride) per milliliter of PRGF.

### PRGF-Endoret infiltration technique

A mild sedation was performed intravenously by an anesthetist with a combination of 2.5 mg of midazolam hydrochloride (5 mg/5 ml, Normon® Laboratories, SA, Madrid, Spain) and 3.2 mg/kg of fentanyl citrate (0.05 mg/ml, Fentanest ®, Kern Pharma SL, Barcelona, Spain) in 100 ml of physiological saline. Additionally, depending on the duration of the procedure, a single dose or repeated doses of 1–2 mg/kg propofol (Propofol® Lipuro 10 mg/ml (1%), B. Braun® Medical S.A., Barcelona, Spain) are administered. The sedation is only performed in the operating room (first and second infiltrations), not in the outpatient clinic (third infiltration).

First, continuous fluoroscopic-guided (Ziehm Solo, Ziehm Imaging GMBH, Germany) intradiscal, facet joint, percutaneous periradicular disc, and peridural infiltration procedures [13] between lumbar L1 and sacral S1 levels were performed in an operating room.

**Fig. 2:**
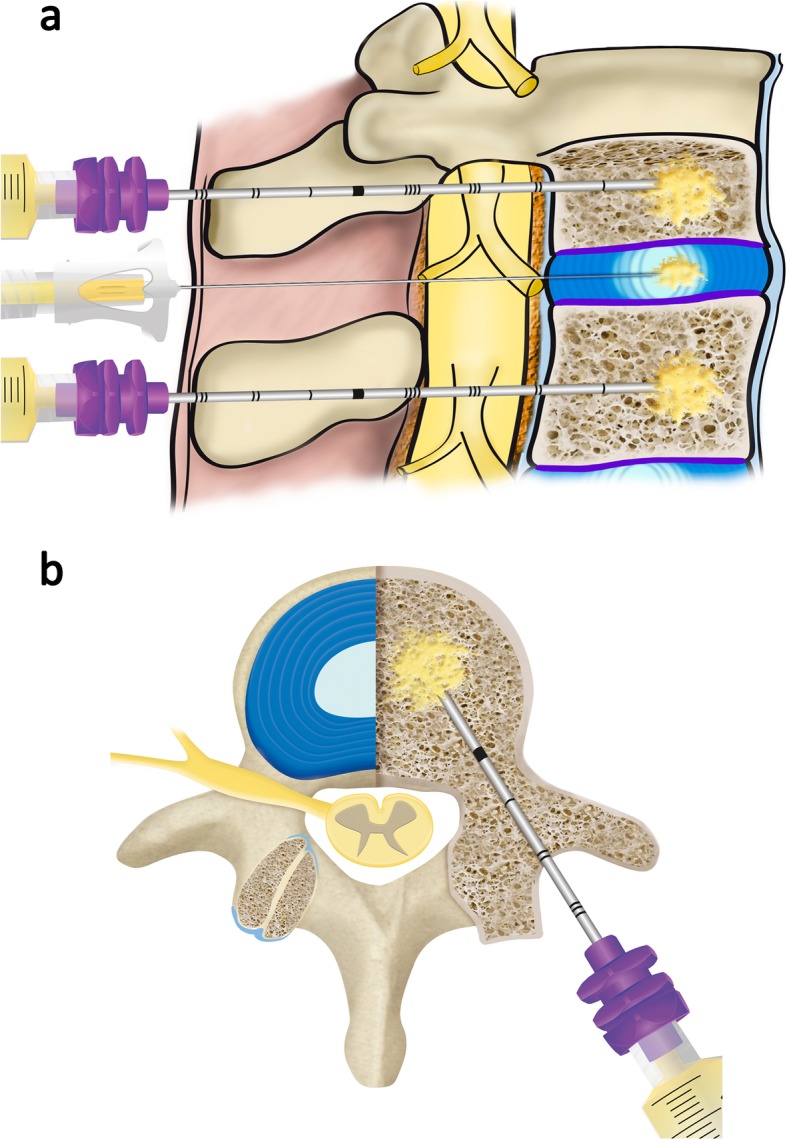
Illustration of the technique used for the intraosseous infiltration of PRGF. **a** Sagittal view. When the disc lesion is adjacent to two injured endplates with Modic type I or II lesions, Schmörl hernias, or fracture sequels [27], the regeneration of the disc lesion is more effective, faster, and safer while simultaneously performing an intraosseous infiltration in the two vertebral bodies adjacent to the disc. Only 4–5 ml of PRGF (F2) are infiltrated and with no need for the tip of the needle to be near the endplate, since this amount fills the entire vertebral body. **b** Axial view showing the transpedicular approach of the needle (15 G, 1.8 mm × 90 mm) that will finally reach the intraosseous level of the vertebral body barely past 1 cm from the back wall of itself. The trocar-biopsy needle system is placed with a low speed power driver

Vertebral intraosseous PRGF infiltrations were completed at the L3 portal (only left side) and L4 vertebral sections. The following description corresponds to the L3 infiltration in L3. A small skin incision was made at the entry point of a biopsy-trocar wire positioned over the bull eye view of the left side of the patient, in a 20–25° oblique fluoroscopic position, until the image of the Scotty dog sign was clearly seen. Subsequently, a 15-G trocar-biopsy needle system (1.8 mm-diameter × 90 mm-length, ARROW® OnControl® Aspiration needle set, Teleflex Medical Europe Ltd., Dublin, Ireland) was introduced and, while leaning on the dog’s eye, 2 ml of local anesthetic (20 mg/ml hydrochloride lidocaine, B. Braun® Medical S.A, Barcelona, Spain) solution was injected to anesthetize the deep soft tissues and the periosteum in the vertebrae where the trocar set is inserted. After waiting for 1 min, the trocar was adjusted to a power driver (Arrow® OnControl® Powered Bone Access System, Teleflex Medical Europe Ltd., Dublin, Ireland). Under lateral fluoroscopic guidance, the trocar was advanced through the pedicle at the junction of the upper third and the middle third of the vertebral body until it was located within the middle of the vertebral body. Subsequently, the power driver was removed and a syringe containing 4–5 ml of sterile activated PRGF F2 was attached to the trocar, liquid that was then gradually and totally injected in the vertebral body (Fig. 2). With this amount of PRGF, the whole vertebral body is replenished, including both endplates. After infiltration, it is necessary to wait 2 min for adequate PRGF coagulation to avoid backward solution migration via the transpedicular demarcation made by the trocar tract. After this, the trocar is removed.

The same vertebral intraosseous PRGF injection procedure was repeated in L4 vertebral subchondral level to stimulate proper tissue regeneration after PRGF infiltrations in both levels.

The patient was then monitored in the recovery room of the ambulatory surgical facility for 1–2 h to observe for any adverse reactions. An ice pack was held over the lumbar injection area to avoid inflammation, and 100 ml physiological saline solution containing analgesic and anti-inflammatory drug (75 mg diclofenac sodium solution 75 mg/3 ml, Llorens® Laboratories, Barcelona, Spain) was administered intravenously to reduce pain (traumatic effect of punctures) and any adverse reactions after the procedure. This medication does not affect the treatment, since it has been administered after the extraction of blood and the PRGF treatment, and only provides short-term pain relief.

One month later, a second application was performed in the operation room following the same procedure as described above.

Four months later, a third dose of PRGF was completed bilaterally at the L4/L5 and L5/S1 levels in an adapted outpatient operating room. This application was not intraosseous but at the deep paravertebral level (lamina). In such a way, the patient was maintained in the prone position. A 25-G Spinocan spinal needle (0.5 mm-diameter and 88 mm-length, B. Braun® Medical S.A, Barcelona, Spain) was inserted vertically, and in parallel, to 1 cm on both sides of the spinous process. The operator guided the depth of needle insertion into the spine until the bone was felt and the lamina or articular process was achieved. After advancing the spinal needle into each vertebral region, 3 ml of sterile activated PRGF was injected in each of both sides of the back of the lumbar vertebrae. After the spinal injection, the patient recovered and was observed for 30 min in the same procedure room.

Physical therapy and other exercises were postponed for 1 week following any of the three procedures described above (first treatment, 1 and 4 months later). The patient experienced no complications in any of the procedures.

## Results

### Evaluation and follow-up

**Fig. 3:**
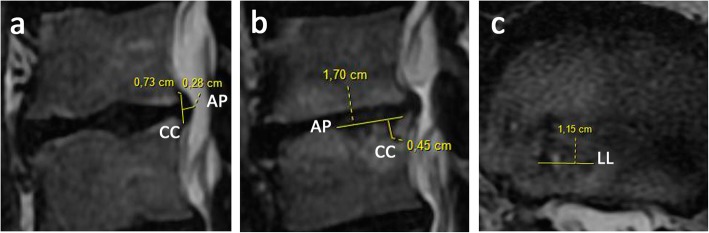
Axial and sagittal T2-weighted images (T2WI) 6 months post-treatment at the lumbar spinal level L3/L4 showing MRI measurements (in mm). **a** Sagittal measurements of lumbar disc protrusion diameter at cranio-caudal (CC) and antero-posterior (AP) sections; **b** sagittal dimension of SND at cranio-caudal (CC) and antero-posterior (AP) sections; and **c** axial dimension of SND at latero-lateral (LL) section

After treatment, the patient’s pain and disability were significantly reduced. Thus, the values were zero for both the NRS and COMI Score. ODI fell to 2% (minimal disability). Follow-up MRI scans (Fig. 3) 6 months after the first PRP treatment revealed unchanged global disc dehydration, degeneration grade at the L3-lower and L4-upper levels, osteochondrosis, TEPS, and Modic changes compared to the pre-procedural lumbar examination (Table 1). Slight regression of the protrusion diameter (PD) at the L3/L4 IVD (11% at the cranio-caudal (CC) axis) was observed. No evidence of compression of the thecal sac or nerve root was noted. Modest SNs were found at the level of intravertebral PRGF infiltrations, showing a significant reduction of the SN diameter (SND) at the L3-lower EP level (61% sagittal CC, 42% axial latero-lateral (LL), and unmeasured sagittal antero-posterior (AP) sections) and at the L4-upper EP level (24% axial LL, 36% sagittal AP, and insignificant changes at the sagittal CC sections).

**Fig. 4:**
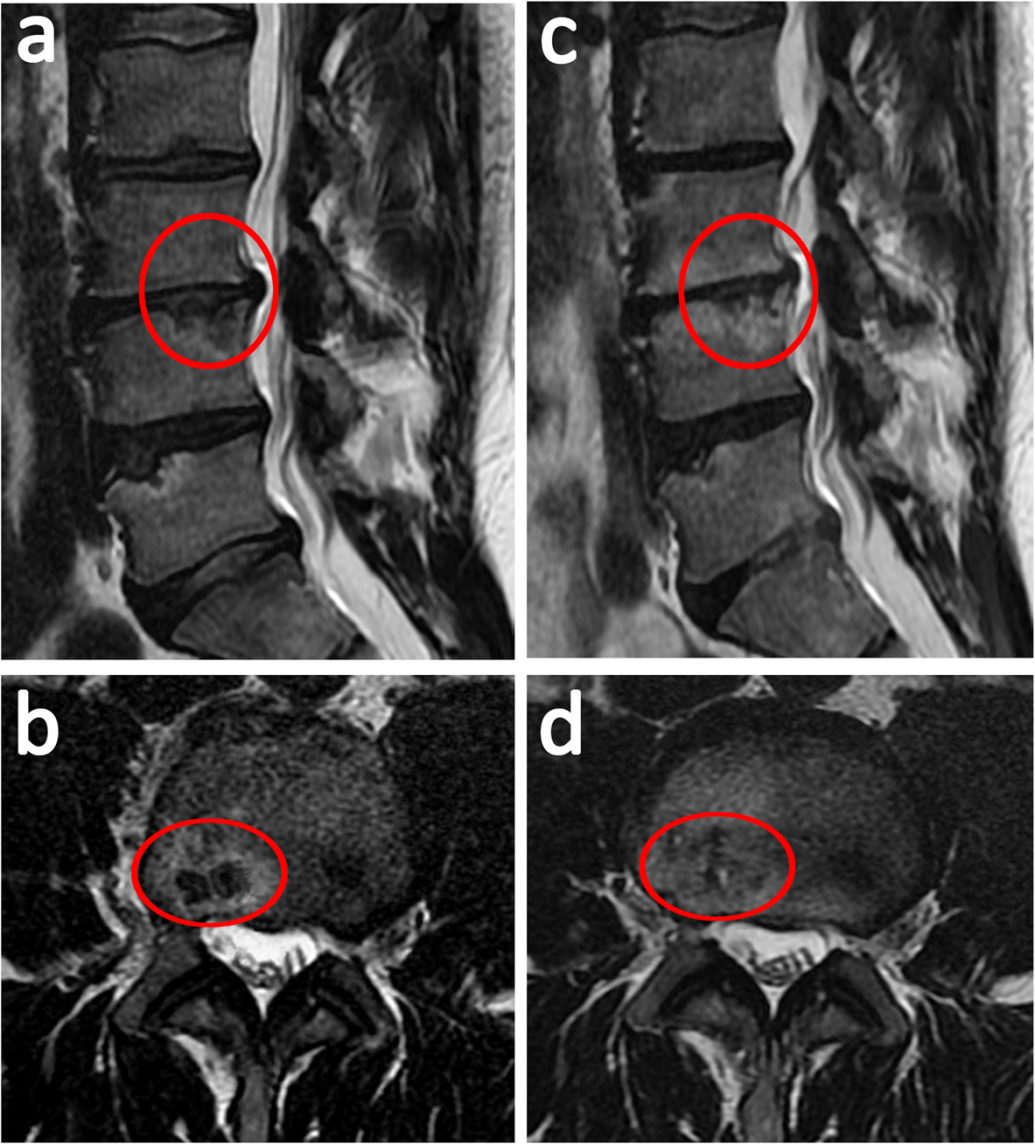
MRI scan before and after platelet-rich plasma treatment. Initial MRI **a** sagittal and **b** axial T2-weighted images (T2WI) (in yellow letters). Six months later, a substantial reduction of Schmörl’s node diameter (SND) was shown on **c** sagittal and **d** axial T2WI at the lumbar spinal level L3/L4. Red circles delineate SN size

Table 1 summarizes the measurements and findings from the MRI analyses of the patient before (Fig. 2) and after (Fig. 3) PRGF treatment. Figure 4 shows the overall comparison between baseline and final MRI.

## Discussion

Discs and endplate regions of the lumbar spinal functional units are particularly affected by biochemical and functional age-related changes, with subsequent disc degeneration.

To date, limited scientific literature exists regarding the long-term effect of PRP treatment and the structural spinal regenerative process [12, 14, 16]. In recent years, PRP infiltrations have been used as an alternative to canonical surgical management to treat DDD and lumbar pain [10–15]. To the best of our knowledge, the combined intra-articular and intraosseous PRP infiltrations have been only reported in patients with knee [18] and hip [19] osteoarthritis for the treatment of chronic pain. In fact, the same approach can be addressed with the intervertebral joint functional unit, as Padilla et al. postulate [5].

A well-known phenomenon of spontaneous regression of herniated lumbar disc material has been extensively documented, but the exact mechanism responsible for the regression of herniated IVD remains poorly understood. On the other hand, limited literature is available regarding resorption of intravertebral herniated discs, such as SNs [7].

Only a few reports demonstrate MRI improvements in disc degeneration [12, 14], such as reduction in disc herniation [12] after PRP treatment. As MRI provides more detailed information concerning intradiscal and intravertebral herniations, the case presented here shows for the first time appreciable MRI improvements in terms of SN regression (Fig. 4, Table 1) after 6 months of PRGF therapy. As a biological strategy developed for the regeneration of spinal structures and pain control, PRGF therapy may contribute to understanding the mechanisms underlying the regression of protruded discs and intravertebral herniations [5].

This novel approach illustrates the need to use standardized and accurate imaging and measurement techniques to evaluate improvements in the lumbar spine in order to correlate improvements in pain and functional outcomes with regeneration of paravertebral structures [28, 29].

Based on our clinical results, we suggest that the process of disc degeneration in our patient was somehow reversed by PRGF infiltrations. PRGF-Endoret is an entirely autologous platelet-rich plasma system that mimics the physiological repair process by releasing autologous growth factors (GFs) and creating a transient biological matrix [30]. The potential mechanisms by which this novel therapeutic combination of intradiscal and vertebral intraosseous PRP infiltrations might affect the progress of DDD or regenerate spinal structures require further elucidation. However, it is reasonable to consider that PRP treatment could stimulate the endogenous repair machinery and induce healing of damaged spinal components [6, 31]. Effects on cell survival and proliferation (induced by GFs), extracellular matrix synthesis (upregulating the production of key matrix proteins), anti-inflammatory mechanisms (downregulating pro-inflammatory cytokines), analgesia (suppression of tumor necrosis factor κB pathways and endogenous cannabinoid systems), subchondral bone homeostasis, and bone mineralization could be produced in a similar manner as described in knee subchondral bone [18], to ultimately restore disc and vertebral bone homeostasis [5]. On the other hand, an improvement in the functional anatomy of the EP would involve restoring the adequate supply of nutrients from the subchondral bone to the IVD [6].

In order to obtain the best clinical results, we have used PRGF-Endoret, a leukocyte-free PRP (P-PRP) comprehensively characterized and classified [32] with a trajectory of more than 20 years [33]. Current evidence suggests that P-PRP may be the most appropriate therapeutic option [34], since the inclusion of leukocytes or erythrocytes in the PRP formulation would not provide any additional advantage, or could even be detrimental to the clinical outcome [35, 36].

## Conclusions

To the best of our knowledge, we present the first reported case description of the utilization of VIO and ID PRP infiltrations to treat protruded discs and intravertebral herniations with a successful clinical outcome. Nonetheless, a clear limitation of this novel biological approach is the description of the technique in a single patient. Additional case reports and clinical studies using this minimally invasive strategy would be necessary to support our preliminary clinical and structural regenerative results.

## Data Availability

Not applicable

## Abbreviations

AP: Antero-posterior
CC: Cranio-caudal
CEP: Cartilage endplate
DDD: Disc degenerative disease
EP: Endplate
GFs: Growth factors
ID: Intradiscal
IVD: Intervertebral disc
K-wire: Kirschner wire
LL: Latero-lateral
MC: Modic changes
MRI: Magnetic resonance imaging
PD: Protrusion diameter
PP: Postero-paramedial
P-PRP: Leukocyte-free PRP
PRGF: Plasma rich in growth factors
PRP: Platelet-rich plasma
SN: Schmörl’s nodes
SND: SN diameter
T2W: T2-weighted
TEPS: Total endplate damage score
VIO: Vertebral intraosseous
VSB: Vertebral subchondral bone
